# C3-Epimer Inclusion Influences Vitamin D Deficiency Classification Near Diagnostic Thresholds in Early Childhood

**DOI:** 10.3390/nu18081188

**Published:** 2026-04-10

**Authors:** Tworowska Julia, Goryński Krzysztof, Goryńska Paulina Zofia, Stampor-Bednarska Justyna, Sobieska-Poszwa Ola, Krogulska Aneta, Siódmiak Joanna, Kowalski Konrad

**Affiliations:** 1 Department of Paediatrics, Allergology and Gastroenterology, Ludwik Rydygier Collegium Medicum Bydgoszcz, Nicolaus Copernicus University Torun, 85-094 Bydgoszcz, Poland; 2 Faculty of Chemical Technology and Engineering, Bydgoszcz University of Science and Technology, Seminaryjna 3, 85-326 Bydgoszcz, Poland; 3 Department of Laboratory Medicine, Faculty of Pharmacy, Ludwik Rydygier Collegium Medicum Bydgoszcz, Nicolaus Copernicus University Torun, 85-094 Bydgoszcz, Poland; 4 Masdiag Sp. z o.o., 01-882 Warsaw, Poland

**Keywords:** vitamin D deficiency, C3-epimer, 25(OH)D, diagnostic threshold, misclassification, LC–MS/MS, early childhood, pediatric nutrition

## Abstract

**Background**: Vitamin D deficiency in early childhood is commonly defined using fixed 25(OH)D thresholds, most often <20 ng/mL. However, inclusion of the C3-epimer (3-epi-25(OH)D_3_) in total 25(OH)D measurements may influence classification, particularly in children with borderline concentrations. **Methods**: In this cross-sectional study of 128 children aged 0–36 months, vitamin D metabolites were quantified using LC–MS/MS in dried blood spot (DBS) samples. Vitamin D deficiency was defined as <20 ng/mL. Total 25(OH)D was recalculated after subtraction of the C3-epimer to assess changes in deficiency classification. Agreement was evaluated using Cohen’s κ, and systematic differences were tested with McNemar’s test. Diagnostic performance parameters were calculated using epimer-resolved 25(OH)D as the reference standard. **Results**: Mean 25(OH)D_3_ concentration was 20.3 ± 6.2 ng/mL, and 57% of children were classified as deficient. After epimer subtraction, deficiency classification changed in 22 of 128 children (17.2%). Agreement between classifications was substantial (κ = 0.67), but McNemar’s test demonstrated a significant systematic shift (*p* < 0.001). Sensitivity of total 25(OH)D including the epimer for detecting deficiency was 70.3% (95% CI: 59.0–80.0%), with specificity of 100% (95% CI: 94.3–100%). Reclassification was strongly concentrated among children with borderline 25(OH)D_3_ concentrations (18–22 ng/mL), where 54.5% were reclassified compared with 4.2% outside this range. Reclassification was not associated with age. **Conclusions**: In young children, inclusion of the C3-epimer in total 25(OH)D measurement leads to potentially clinically relevant misclassification of vitamin D deficiency, particularly near diagnostic thresholds. Epimer-resolved assessment may improve diagnostic precision in cases with borderline vitamin D concentrations.

## 1. Introduction

Vitamin D deficiency in early childhood is commonly defined using circulating 25-hydroxyvitamin D [25(OH)D] thresholds, most frequently <20 ng/mL, as recommended by several clinical guidelines, although different organizations have proposed varying thresholds for defining vitamin D deficiency [1,2]. The choice of threshold may influence the classification of vitamin D status and, consequently, the interpretation of analytical differences. In pediatric practice, decisions regarding supplementation and follow-up frequently rely on fixed cut-off values. However, interpretation of vitamin D status is influenced by both biological and analytical factors that may affect measured 25(OH)D concentrations [3,4].

One important biological factor in early life is the presence of the C3-epimer of 25(OH)D_3_ (3-epi-25(OH)D_3_). The proportion of this epimer is higher in infants and young children compared with older populations [5,6]. This phenomenon is thought to reflect developmental differences in vitamin D metabolism, including increased activity of C3-epimerization pathways and immaturity of hepatic enzyme systems in early life. In addition, higher relative vitamin D intake per body weight in infancy may contribute to enhanced formation of the epimer. Although 3-epi-25(OH)D_3_ can bind to the vitamin D receptor, its biological activity appears reduced, and its clinical significance remains incompletely understood [6,7]. However, as the biological activity of the C3-epimer is reduced but not completely absent, subtraction of this metabolite may potentially lead to underestimation of functionally available vitamin D. Therefore, the present analysis should be interpreted as an assessment of analytical impact on classification rather than a direct reflection of biological vitamin D status. Importantly, many routine immunoassays do not distinguish the epimer from 25(OH)D_3_, potentially leading to overestimation of total 25(OH)D concentrations when epimer-resolved methods are not used [4,5,7]. DBS sampling offers a minimally invasive alternative to venous blood collection and is particularly suitable for pediatric populations.

Liquid chromatography–tandem mass spectrometry (LC–MS/MS) allows separate quantification of 25(OH)D_3_ and 3-epi-25(OH)D_3_ and is increasingly regarded as the analytical reference method for vitamin D assessment [3,8]. Previous studies have described age-related differences in epimer proportions and emphasized analytical considerations in pediatric populations [5,6,9]. However, data addressing the direct clinical impact of epimer inclusion on vitamin D deficiency classification at established diagnostic thresholds remain limited.

This issue may be particularly relevant in children with 25(OH)D concentrations near diagnostic cut-offs. Small differences in measured total 25(OH)D may shift individuals across the <20 ng/mL threshold, potentially leading to misclassification of deficiency. Previous studies have described the presence, proportion, and age-related variability of C3-epimers in infants and young children [5,6,9]. However, the direct impact of C3-epimer inclusion on vitamin D deficiency classification at clinically used diagnostic thresholds has not been systematically evaluated [10,11].

Therefore, the primary aim of this study was to assess whether inclusion of the C3-epimer affects vitamin D deficiency classification in children aged 0–3 years, with particular focus on concentrations near the diagnostic threshold. Secondary objectives included evaluation of agreement between epimer-resolved and non-epimer-resolved classifications and estimation of diagnostic performance parameters.

## 2. Materials and Methods

### 2.1. Study Design and Setting

This was a prospective, cross-sectional study conducted between 2016 and 2019 at the Department of Paediatrics, Allergology and Gastroenterology, Ludwik Rydygier Collegium Medicum Bydgoszcz, Nicolaus Copernicus University, a tertiary pediatric center located in Bydgoszcz, Poland. Participants were children from birth to 3 years who underwent inpatient diagnostic evaluation for allergic diseases, congenital renal anomalies, or feeding difficulties. This population represents a clinically referred cohort rather than a general population sample.

### 2.2. Study Population and Group Allocation

A total of 128 children aged 0–3 years who met all inclusion criteria and had complete datasets were included in the final analysis. Participants who did not meet eligibility criteria or had incomplete data were excluded prior to analysis. All participants were hospitalized for diagnostic evaluation due to allergic diseases, congenital renal anomalies, or feeding difficulties. The patient baseline characteristics are summarized in Table 1.

### 2.3. Inclusion and Exclusion Criteria

Inclusion criteria:

- Age 0–3 years;
- Clinical assessment at the study site during the recruitment period;
- Availability of a 3-day dietary record;
- Availability of a dried blood spot (DBS) sample.

Exclusion criteria:

- Chronic liver disease;
- Chronic kidney disease stage ≥ 3;
- Disorders of calcium–phosphate metabolism other than suspected CYP24A1;
- Celiac disease;
- Other malabsorption syndromes;
- Insufficient sample quality or quantity for LC–MS/MS analysis.

### 2.4. Clinical Assessment and Questionnaire

Parents/guardians completed a self-designed questionnaire comprising 10 open-ended items and 20 closed-ended questions. The instrument captured: demographics; perinatal history; diet (including fortified products and fish intake); vitamin D supplementation (dose, formulation, schedule, adherence); medical history (allergic, renal, gastrointestinal); current medications; and symptoms suggestive of calcium–phosphate disturbances. Anthropometrics (weight, length/height, head circumference) were measured by trained staff using standardized procedures; z-scores were calculated with WHO guidelines [12].

Usual dietary vitamin D intake was estimated from a 3-day food record (two weekdays and one weekend day). Parents were instructed by a dietitian and provided portion-size guides. Records were reviewed for completeness and analyzed with DIETA 5 nutritional analysis software (National Institute of Public Health, Kraków, Poland). Average daily vitamin D intake (IU/day and µg/day) was computed; intake from supplements was recorded separately. For conversion, 1 µg of vitamin D corresponds to 40 IU.

### 2.5. Vitamin D Sampling and Measurement

Capillary blood was collected via finger or earlobe puncture onto standardized filter paper cards (dried blood spots, DBS) using a commercially available self-sampling kit. Cards were dried for ≥3 h at ambient temperature, stored with desiccant, and transported to the central laboratory.

From each card, two 3 mm discs (corresponding to approximately 6 µL of blood) were punched and transferred to a 96-well plate. Vitamin D metabolites were extracted using 150 µL of methanol containing deuterated internal standards (d_6_-25(OH)D_3_, d_3_-3-epi-25(OH)D_3_, d_3_-25(OH)D_2_, d_6_-24,25(OH)_2_D_3_). The extracts were shaken for 30 min at room temperature and centrifuged, and the supernatant was evaporated under a gentle stream of nitrogen at 50 °C.

Subsequently, 4-(4′-dimethylaminophenyl)-1,2,4-triazoline-3,5-dione (DAPTAD) derivatization was performed by adding 60 µL of reagent solution (200 µg mL^−1^ in ethyl acetate) and incubating for 30 min. Residues were reconstituted in methanol/water (1:1, *v*/*v*), and 20 µL aliquots were injected into the LC–MS/MS system.

Chromatographic separation was performed using a high-performance liquid chromatography system coupled to a triple quadrupole mass spectrometer. Separation was carried out on a Kinetex F5 column (1.7 µm, 50 × 2.1 mm; Phenomenex, Torrance, CA, USA) at 40 °C with a mobile phase consisting of water and acetonitrile (both containing 0.1% formic acid) at a flow rate of 0.45 mL min^−1^. Detection was performed using a triple quadrupole mass spectrometer equipped with an electrospray ionization (ESI) source operating in positive ion mode, with multiple reaction monitoring (MRM) transitions optimized for each analyte. Quantified metabolites included 25(OH)D_3_, 3-epi-25(OH)D_3_, 25(OH)D_2_, and 24,25(OH)_2_D_3_.

Calibration curves were prepared using fortified artificial serum (2% human serum albumin in phosphate buffer) mixed 1:1 with red blood cell concentrate to simulate whole blood. The analytical method was validated in accordance with FDA bioanalytical guidelines, including assessment of linearity, precision, accuracy, sensitivity, and stability. The proposed sample preparation protocol was assessed following a previously published procedure [8], with the equivalence between serum and DBS determinations confirmed in a comparative study using samples obtained from healthy volunteers. All measurements in the present study were performed using DBS samples; no serum measurements were used. The analytical performance characteristics of the method, including precision (CV%), accuracy, and recovery for individual vitamin D metabolites, have been previously reported for this DBS-based LC–MS/MS approach. Study-specific precision parameters were not reassessed, as the validated analytical protocol was applied without modification [8].

### 2.6. Outcomes and Definitions

Primary outcome: change in vitamin D deficiency classification (<20 ng/mL) after subtraction of the C3-epimer.

Secondary outcomes included concentrations of individual vitamin D metabolites and derived indices, including the vitamin D metabolite ratio (VMR) and the percentage of C3-epi-25(OH)D_3_ (%C3-epi-25(OH)D_3_), as well as their associations with dietary, anthropometric, and clinical variables. The VMR was calculated as 25(OH)D_3_/24,25(OH)_2_D_3_. High VMR was defined as values above the cohort median.

### 2.7. Ethical Considerations

The protocol was approved by the Local Bioethics Committee (389/2017, 13 June 2017). Written informed consent was obtained from parents/legal guardians of all participants.

### 2.8. Statistical Analysis

Categorical variables are presented as counts (*n*) and percentages; continuous variables as mean ± SD or median (IQR), as appropriate. Normality of distribution was assessed using the Shapiro–Wilk test. Group comparisons were performed using the χ^2^ or Fisher’s exact test for categorical variables and Student’s *t*-test or Mann–Whitney U test for continuous variables, depending on data distribution. Correlations were examined using Pearson’s or Spearman’s coefficients, as appropriate.

Multiple linear regression models were constructed to evaluate independent predictors of vitamin D metabolite concentrations and derived indices. Covariates were selected based on clinical relevance and assessment of multicollinearity (variance inflation factor < 5).

Seasonal variation was evaluated according to the month of blood collection and categorized into meteorological seasons (winter: December–February; spring: March–May; summer: June–August; autumn: September–November). Differences in 25(OH)D_3_, %C3-epimer, and vitamin D metabolite ratio (VMR) across seasons were assessed using the Kruskal–Wallis test due to non-normal distribution in seasonal subgroups. Differences in vitamin D deficiency prevalence (<20 ng/mL) between seasons were analyzed using the χ^2^ test.

To evaluate the clinical impact of C3-epimer inclusion, total 25(OH)D was recalculated after subtraction of 3-epi-25(OH)D_3_. Vitamin D deficiency was defined as <20 ng/mL for both epimer-inclusive and epimer-resolved calculations.

Agreement between classifications with and without epimer inclusion was assessed using Cohen’s kappa coefficient (κ). Systematic differences between paired classifications were evaluated using McNemar’s test for dependent proportions.

Diagnostic performance of total 25(OH)D including the epimer was assessed using epimer-resolved 25(OH)D as the reference standard. Sensitivity, specificity, positive predictive value (PPV), and negative predictive value (NPV) were calculated.

To examine the distribution of reclassification across concentration ranges, subgroup analysis was performed for children with borderline 25(OH)D_3_ concentrations (18–22 ng/mL). Differences in continuous variables between reclassified and non-reclassified groups were analyzed using the Mann–Whitney U test.

All tests were two-sided, and *p* < 0.05 was considered statistically significant. Statistical analyses were performed using IBM SPSS Statistics v 29. No formal a priori sample size calculation was performed, as all eligible participants during the recruitment period were included. Therefore, the study should be considered exploratory rather than confirmatory. Nevertheless, the primary paired comparison yielded a statistically significant result.

## 3. Results

### 3.1. Study Population

The study included 128 children aged 0–36 months (mean 14.1 ± 9.5 months), of whom 57 (44.5%) were female. Most participants lived in urban areas (66.4%), and 21.1% were exposed to household secondhand smoke. Cesarean delivery occurred in 46.9% of cases, and 9.4% were born preterm. The mean birth weight was 3395 ± 617 g. Mothers had higher education in 58.6% of cases, and 46.9% of fathers held tertiary degrees (Table 1). Exclusive breastfeeding was reported in 25.8% of participants, predominant formula feeding in 68.7%, and cow’s milk feeding in 5.5%. Data are presented in Table 1.

Mean total daily vitamin D intake was 688.6 ± 551.4 IU, including 249.0 ± 236.1 IU from diet and 439.6 ± 509.2 IU from supplementation (Table 2).

### 3.2. Vitamin D Status and Distribution

**Figure 1:**
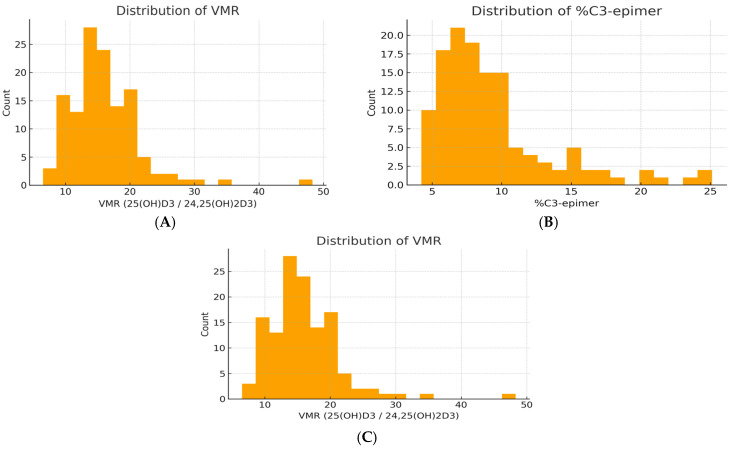
Distribution of vitamin D metabolites in the study population. (**A**) Total 25(OH)D concentration. (**B**) Relative contribution of the C3-epimer. (**C**) Vitamin D metabolite ratio (VMR = 25(OH)D_3_/24,25(OH)_2_D_3_).

Mean 25(OH)D_3_ concentration was 20.3 ± 6.2 ng/mL, and 57% of children were classified as vitamin D deficient (<20 ng/mL). Total 25(OH)D ranged from 10.3 to 46.8 ng/mL, with a median of approximately 19 ng/mL (Figure 1A). All metabolite distributions were right-skewed (Shapiro–Wilk *p* < 0.001) (Figure 1B,C).

### 3.3. Reclassification Analysis

When total 25(OH)D was recalculated after subtraction of the C3-epimer, deficiency classification changed in 22 of 128 children (17.2%). In all reclassified cases, inclusion of the epimer resulted in apparent vitamin D sufficiency.

Agreement between classifications with and without epimer inclusion was substantial (Cohen’s κ = 0.67). However, McNemar’s test demonstrated a significant systematic difference between methods (*p* < 0.001).

Using epimer-resolved 25(OH)D as the reference standard, classification based on total 25(OH)D including the epimer showed: sensitivity: 70.3% (95% CI: 59.0–80.0%), specificity: 100% (95% CI: 94.3–100%).

Nearly 30% of children classified as sufficient when including the epimer were deficient after epimer subtraction.

**Figure 2:**
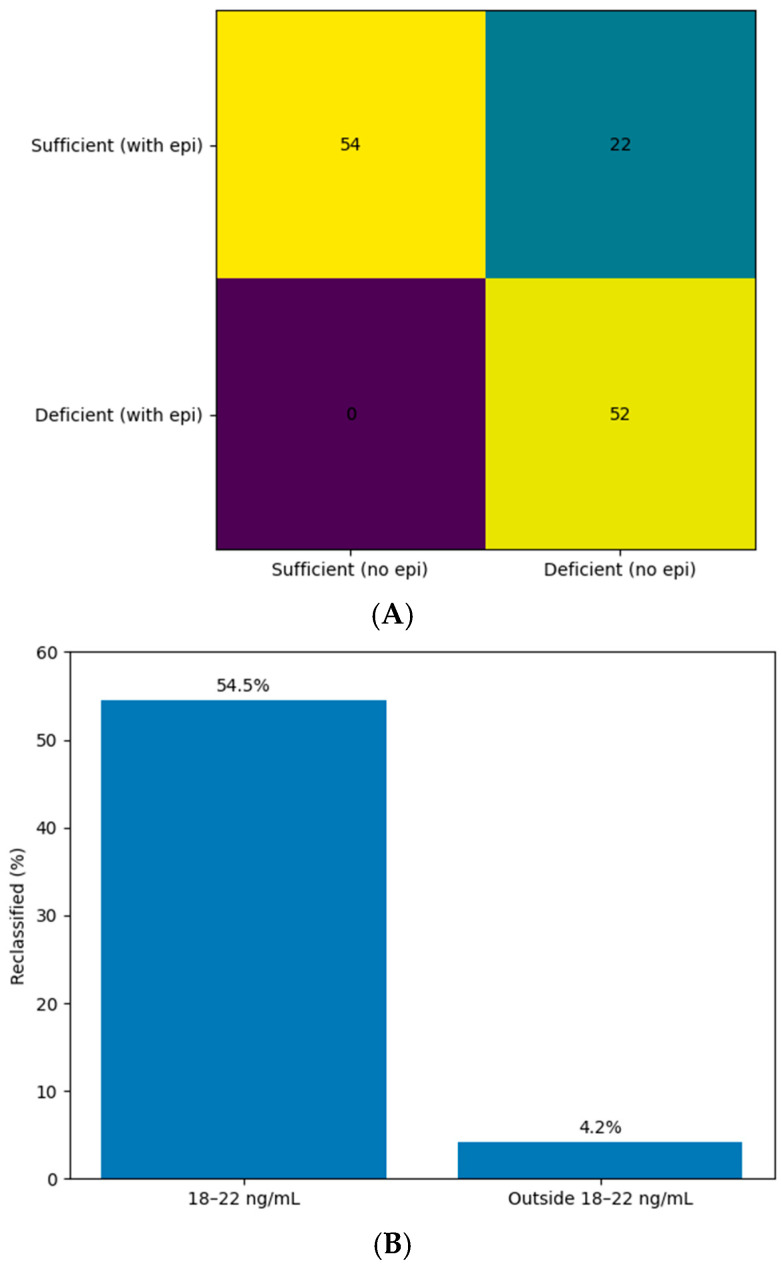
Impact of C3-epimer inclusion on vitamin D deficiency classification. (**A**) Cross-classification of vitamin D deficiency status (<20 ng/mL) with and without inclusion of the C3-epimer. Values represent the number of children in each classification category. Cohen’s κ = 0.67. McNemar’s test indicated a significant systematic difference between classifications (*p* < 0.001). (**B**) Proportion of children reclassified after epimer subtraction according to baseline 25(OH)D_3_ concentration. Reclassification occurred in 54.5% of children with borderline concentrations (18–22 ng/mL) compared with 4.2% among those outside this range.

Reclassification was strongly concentrated in children with 25(OH)D_3_ concentrations in the borderline range of 18–22 ng/mL. In this subgroup, 54.5% were reclassified, compared with only 4.2% among children outside this range (Figure 2A,B). Notably, reclassification was not limited to the borderline range. Among the 22 reclassified children, 10 (approximately 45%) had baseline 25(OH)D_3_ concentrations above 22 ng/mL, indicating that the impact of C3-epimer inclusion may extend beyond the immediate vicinity of the diagnostic threshold.

Reclassification was not associated with age (Mann–Whitney *p* = 0.89) and occurred at similar proportions in infants ≤ 6 months (19.5%) and older children.

### 3.4. Age-Related Variability in Vitamin D Metabolism

**Figure 3:**
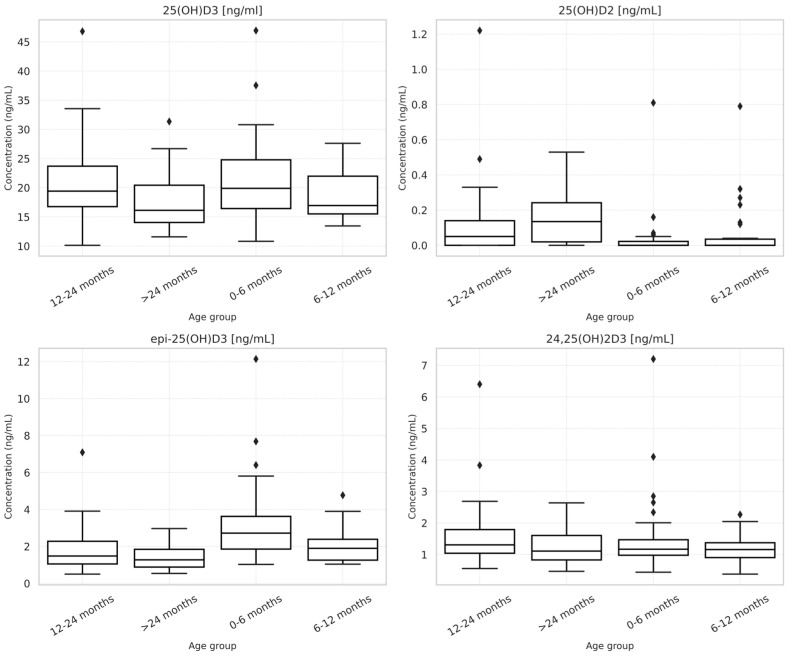
Age-related differences in serum vitamin D metabolites.

Metabolite concentrations differed by age group (Table 3, Figure 3). Infants aged 0–6 months had the highest 3-epi-25(OH)D_3_ concentrations (mean 3.26 ± 2.21 ng/mL, *p* < 0.001). In contrast, 25(OH)D_2_ increased with age (*p* < 0.001), likely reflecting introduction of fortified foods. No significant age-related differences were observed for 25(OH)D_3_ or 24,25(OH)_2_D_3_.

Additionally, C3-epi-25(OH)D_3_ concentrations tended to be higher in children born via vaginal delivery compared with those born by cesarean section (2.45 vs. 1.90 ng/mL); however, this difference did not reach statistical significance (*p* = 0.074).

### 3.5. Predictors of Vitamin D Deficiency and Metabolite Indices

In multivariable logistic regression (Table 4), a binary indicator of maternal illness during pregnancy independently predicted vitamin D deficiency (OR 2.29, 95% CI 1.02–5.16) and high VMR (OR 2.79, 95% CI 1.15–6.78). Younger age was associated with a higher proportion of C3-epimer (OR 0.90 per month, 95% CI 0.82–1.00). Higher dietary vitamin D intake and greater birth weight independently predicted increased VMR.

**Figure 4:**
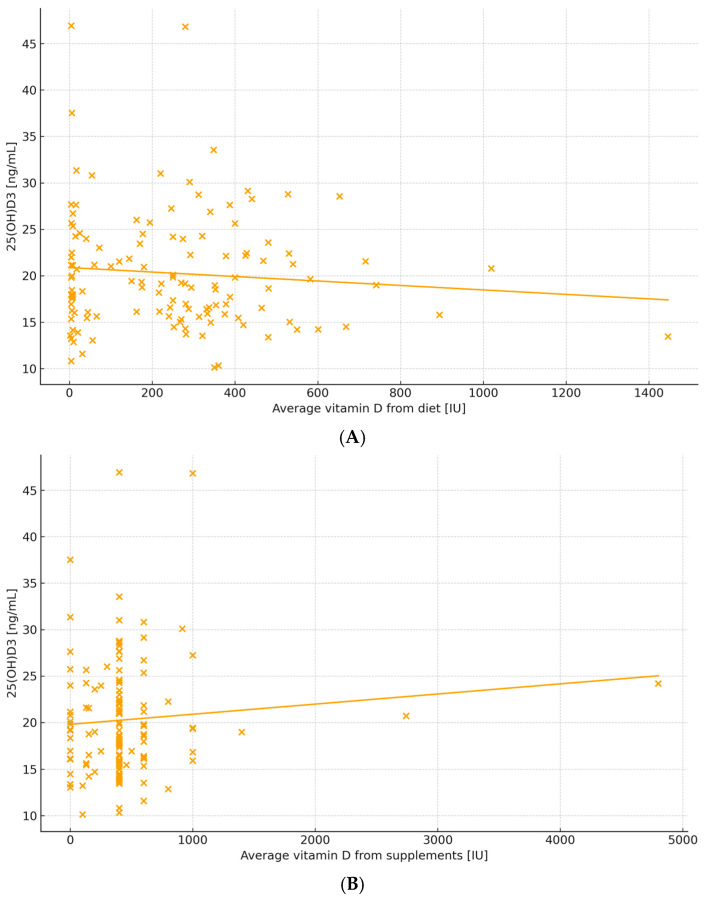
Association between vitamin D intake and serum 25(OH)D_3_ concentration. (**A**) Relationship between daily dietary vitamin D intake (IU/day) and serum 25(OH)D_3_ concentration. No significant correlation was observed (Spearman’s r = −0.10, *p* = 0.34). (**B**) Relationship between daily supplemental vitamin D intake (IU/day) and serum 25(OH)D_3_ concentration. A positive correlation was observed (Spearman’s r = 0.29, *p* = 0.008).

Daily dietary vitamin D intake was not associated with serum 25(OH)D_3_ (r = −0.10, *p* = 0.34), whereas supplementation showed a positive correlation (r = 0.29, *p* = 0.008) (Figure 4A,B).

### 3.6. Seasonal Variation

No significant seasonal differences were observed for 25(OH)D_3_ (Kruskal–Wallis *p* = 0.066), %C3-epimer (*p* = 0.062), or VMR (*p* = 0.753). Vitamin D deficiency prevalence did not differ between seasons (χ^2^ *p* = 0.191).

## 4. Discussion

In this cross-sectional study of 128 infants and young children (0–36 months), we demonstrate that inclusion of the C3-epimer in total 25(OH)D measurement leads to potentially clinically relevant misclassification of vitamin D deficiency. Seventeen percent of children were reclassified after epimer subtraction, and agreement between classifications was only substantial (κ = 0.67), not perfect. McNemar’s test confirmed a systematic shift in classification (*p* < 0.001), indicating that epimer inclusion shifted classification toward apparent sufficiency in this cohort.

Importantly, the effect was strongly concentrated in children with 25(OH)D_3_ concentrations near the diagnostic threshold. More than half of children in the borderline range (18–22 ng/mL) were reclassified after epimer subtraction, whereas misclassification was less frequent outside this range. However, reclassification was not limited exclusively to borderline cases. A substantial proportion of reclassified children had baseline 25(OH)D_3_ concentrations above 22 ng/mL, indicating that the impact of C3-epimer inclusion may extend beyond the immediate vicinity of the diagnostic threshold.

These findings indicate that epimer-related distortion primarily affects individuals around clinically relevant decision thresholds but may also influence classification in individuals who would otherwise be considered sufficient. In clinical practice, where decisions often hinge on fixed thresholds such as <20 ng/mL, this phenomenon may result in underdiagnosis of deficiency and has direct practical implications. Using epimer-resolved 25(OH)D as the reference standard, total 25(OH)D including the epimer demonstrated a sensitivity of only 70.3% for detection of deficiency, although specificity remained 100%. Nearly one-third of children classified as sufficient when including the epimer were deficient after subtraction. This reduction in diagnostic sensitivity highlights the clinical importance of epimer-resolved assessment, particularly in pediatric populations where epimer fractions are higher than in adults. It should also be noted that the impact of epimer inclusion on classification may vary depending on the diagnostic threshold used, as different guidelines propose different cut-offs for vitamin D deficiency. However, routine implementation of LC–MS/MS for all patients may be limited by cost and availability in many clinical settings. A more pragmatic approach could involve the use of conventional assays for initial screening, with epimer-resolved LC–MS/MS reserved for cases with borderline 25(OH)D concentrations (e.g., 18–22 ng/mL), where the risk of misclassification is highest. Such a targeted strategy may improve diagnostic accuracy while maintaining feasibility in routine clinical practice. LC–MS/MS-based measurement of vitamin D metabolites, including epimer-resolved analysis, is available in specialized clinical and reference laboratories. However, its broader implementation in routine practice may be limited by higher costs, need for dedicated instrumentation, and technical expertise. In contrast, automated immunoassays remain more accessible and cost-effective for large-scale screening.

Age-related analysis confirmed that epimerization is most pronounced in early infancy, consistent with previous reports [3,7,13]. Reported proportions of the C3-epimer vary substantially across studies depending on age distribution, analytical methods, and whether concentrations are expressed as absolute values or percentages of total 25(OH)D. However, reclassification was not confined to the youngest children and showed no significant association with age. This suggests that misclassification arises primarily from dependence on baseline 25(OH)D status relative to decision limits rather than from extreme epimer proportions in a specific developmental subgroup. This apparent discrepancy suggests that the clinical impact of the C3-epimer is primarily determined by the proximity of total 25(OH)D_3_ concentrations to the diagnostic threshold, rather than by the absolute epimer concentration itself. Although infants aged 0–6 months exhibited higher epimer levels, their total 25(OH)D_3_ concentrations were not significantly lower than in older children, which may have reduced the likelihood of crossing the diagnostic threshold after epimer subtraction. These findings support a threshold-dependent effect, in which analytical variation becomes clinically relevant mainly in individuals with values near established cut-offs.

Although the primary focus of this study was diagnostic reclassification, additional analyses provide context regarding vitamin D metabolism in early life. Higher dietary vitamin D intake and greater birth weight were independently associated with increased VMR, reflecting adaptive upregulation of 24-hydroxylation with greater substrate availability [14,15,16,17]. The wide variability in vitamin D supplementation observed in this cohort likely reflects heterogeneous dosing regimens and adherence patterns in routine pediatric practice. A binary self-reported indicator of maternal illness during pregnancy was associated with vitamin D deficiency and higher VMR. However, this variable was heterogeneous and not specified by diagnosis, limiting its biological interpretability. Therefore, this finding should be interpreted cautiously and considered exploratory, as it may reflect residual confounding rather than a direct pathophysiological relationship. The variable was retained in the multivariable model to account for potential maternal and prenatal influences, but not to support mechanistic conclusions. These findings support the concept that vitamin D metabolism in infancy reflects integrated nutritional and developmental influences. Although not statistically significant, a trend toward higher C3-epimer concentrations in children born via vaginal delivery was observed, which may warrant further investigation in larger cohorts.

No significant seasonal differences were observed for 25(OH)D_3_, %C3-epimer, VMR, or deficiency prevalence. The modest seasonal variation may reflect routine supplementation in this age group. However, because sunlight exposure was not directly quantified and the cohort was not population-based, subtle seasonal effects cannot be excluded.

Recent clinical practice guidelines from the Endocrine Society discourage universal vitamin D screening while endorsing supplementation in high-risk groups [18]. Our data suggest that when vitamin D is measured, particularly in children with borderline concentrations, epimer-resolved assessment may reduce the risk of underdiagnosing deficiency. Rather than abandoning threshold-based interpretation, these findings indicate that analytical specificity becomes most critical near diagnostic cut-offs.

### Limitations

This study has several limitations. Its cross-sectional design precludes causal inference. The study population consisted of hospitalized children undergoing diagnostic evaluation, which introduces potential selection bias and limits generalizability to the general pediatric population. These children may differ in underlying health status, nutritional patterns, and vitamin D metabolism compared with healthy individuals. Therefore, the findings should be interpreted within the context of a clinically referred population. At the same time, this cohort reflects real-world clinical settings in which vitamin D testing is most frequently performed, particularly in children with suspected or complex conditions.

Dietary intake was parent-reported and subject to recall bias, and sunlight exposure was not directly measured. The study did not include biochemical markers of calcium–phosphate metabolism (e.g., PTH, calcium, phosphorus, or bone turnover markers such as ALP), which limits the ability to determine whether reclassified children had functionally significant vitamin D deficiency. Consequently, it cannot be established whether the observed differences represent clinically meaningful hypovitaminosis or primarily reflect an analytical refinement in vitamin D measurement. Future studies incorporating functional biochemical markers are needed to clarify the clinical relevance of epimer-related reclassification.

Although the DBS-based LC–MS/MS method has been previously validated, study-specific precision parameters (e.g., CV%) were not reassessed, which may limit evaluation of analytical variability in the present dataset. In addition, matrix effects and hematocrit variability inherent to dried blood spot analysis may influence measurement accuracy.

The variable “maternal illness during pregnancy” was recorded as a binary self-reported measure without differentiation of diagnoses, limiting mechanistic interpretation. Active vitamin D metabolite (1,25-dihydroxyvitamin D) was not measured, as the study focused on 25(OH)D-related metabolites relevant for deficiency classification.

Finally, no formal a priori sample size calculation was performed. Therefore, the study should be interpreted as exploratory, and future studies with prospectively calculated sample sizes are needed to confirm the magnitude and clinical implications of the observed reclassification effect.

## 5. Conclusions

In young children, inclusion of the C3-epimer in total 25(OH)D measurement leads to potentially clinically relevant misclassification of vitamin D deficiency, particularly among individuals with concentrations near established diagnostic thresholds. Epimer inclusion reduced diagnostic sensitivity to 70% and resulted in reclassification of 17% of participants. These findings indicate that analytical resolution of the C3-epimer may be most important in cases with borderline vitamin D values. Integrating epimer-resolved assessment into pediatric evaluation could improve diagnostic precision without fundamentally altering existing clinical thresholds.

## Figures and Tables

**Table 1:** Demographic, perinatal, and environmental characteristics of the study population (*n* = 128).

Parameter	Study Group *n* = 128 (100%)
Age, months, mean ± SD	14.1 ± 9.5
Min-max	2–36
Sex, *n* (%)	
female	57 (44.5)
male	71 (55.5)
Place of living, *n* (%)	
village	43 (33.6)
city	85 (66.4)
Household secondhand smoke exposure, *n* (%)	
yes	27 (21.1)
no	101 (78.9)
Maternal education, *n* (%)	
primary	19 (14.8)
secondary	34 (26.6)
higher	75 (58.6)
Paternal education, *n* (%)	
primary	21 (16.4)
secondary	47 (36.7)
higher	60 (46.9)
Mode of delivery, *n* (%)	
vaginal	68 (53.1)
cesarean section	60 (46.9)
Birth weight, g, mean ± SD	3395 ± 617.2
Gestational age at birth, *n* (%)	
term	116 (90.6)
preterm	12 (9.4)

**Table 2:** Daily vitamin D intake from diet and supplementation in the study population.

Parameter	Mean ± SD [IU/Day]
Dietary vitamin D intake	249.00 ± 236.14
Vitamin D from supplementation	439.55 ± 509.22
Total vitamin D intake from diet and supplementation	688.56 ± 551.38

**Table 3:** Age-related differences in serum vitamin D metabolite concentrations.

Metabolite	Mean ± SD, ng/mL 0–6 Months	Mean ± SD, ng/mL 6–12 Months	Mean ± SD, ng/mL 12–24 Months	Mean ± SD, ng/mL >24 Months	*p*
25(OH)D_3_	21.61 ± 7.28	18.80 ± 4.24	20.66 ± 6.29	18.29 ± 5.87	0.176
25(OH)D_2_	0.04 ± 0.14	0.07 ± 0.17	0.11 ± 0.19	0.17 ± 0.17	<0.001
3-epi-25(OH)D_3_	3.26 ± 2.21	1.97 ± 0.89	1.77 ± 1.08	1.45 ± 0.72	<0.001
24,25(OH)_2_D_3_	1.54 ± 1.19	1.19 ± 0.44	1.56 ± 0.94	1.27 ± 0.63	0.304

**Table 4:** Multivariable logistic regression analysis identifying predictors of vitamin D deficiency and metabolite indices.

Predictor (Reference)	Vitamin D Deficiency OR (95% CI)	High %C3-Epi OR (95% CI)	High VMR OR (95% CI)
Maternal illness in pregnancy (yes vs. no)	2.29 (1.02–5.16)	Ns	2.79 (1.15–6.78)
Age (months)	Ns	0.90 (0.82–1.00) (per month)	Ns
Dietary vitamin D intake (per 100 IU/day)	Ns	Ns	1.47 (1.12–1.94)
Birth weight (per 100 g)	Ns	Ns	1.15 (1.04–1.26)

## Data Availability

The original contributions presented in this study are included in the article. Further inquiries can be directed to the corresponding author.

## Abbreviations

25(OH)D	25-hydroxyvitamin D
25(OH)D_2_	25-hydroxyvitamin D_2_
25(OH)D_3_	25-hydroxyvitamin D_3_
3-epi-25(OH)D_3_	C3-epimer of 25-hydroxyvitamin D_3_
24,25(OH)_2_D_3_	24,25-dihydroxyvitamin D_3_
VMR	vitamin D metabolite ratio
LC–MS/MS	liquid chromatography–tandem mass spectrometry
DBS	dried blood spots
MRM	multiple reaction monitoring
ESI	electrospray ionization
SD	standard deviation
IQR	interquartile range
PPV	positive predictive value
NPV	negative predictive value
WHO	World Health Organization
